# Cellular Pathology
**Cellular Pathology as based upon Physiological and Pathological Histology.* By Rudolf Virchow, Public Professor in Ordinary of Pathological Anatomy, General Pathology and Therapeutics, in the University of Berlin, &c. Translated from the Second Edition of the Original, by Frank Chance, B.A., M.B., Cantab., L.R.C.P., Physician to the Blenheim Free Dispensary and Infirmary. 8vo. London: Churchill. 1860.


**Published:** 1861-04

**Authors:** 


### Art. XV. CELLULAR PATHOLOGY.*

Dr. Chance has done excellent service to the medical literature of this country by his admirable translation of Professor Virchow's work on *Cellular Pathology*. This important work (dedicated by its distinguished author to Professor Goodsir) consists of a series of lectures, delivered in the early part of 1858, before the medical public of Berlin, in the new Pathological Institute of the University of that city. The object chiefly aimed at in these lectures, the learned Professor states, in the preface to the first edition of the book,—

"—— was to furnish a clear and connected explanation of those facts upon which, according to his ideas, the theory of life must now be based, and out of which, also, the science of pathology has now to be constructed. They were more particularly intended as an attempt to offer, in a better arranged form than had hitherto been done, a view of the cellular nature of all vital processes, both physiological and pathological, animal and vegetable, so as distinctly to set forth what even the people have long been dimly conscious of, namely, the unity of life in all organized beings, in opposition to the one-sided humoral and neuristical (solidistic) tendencies which have been transmitted from the mythical days of antiquity to our own times, and at the same time to contrast with the equally one-sided interpretations of a grossly mechanical and chemical bias, the more delicate mechanism of the cell." (p. vii.)

After briefly setting forth the manner in which he has endeavoured to effect this object, the Professor further expresses a hope, that what he has offered "may not be taken for more than it is intended to be," and adds—

"Those who have found leisure enough to keep up their knowledge by reading the current medical literature, will find but little that is new in these lectures. The rest will not, by reading them, be spared the trouble of being obliged to study the subjects, which are here only briefly touched upon, more closely in the special histological, physiological, and pathological works. But they will at least be in possession of a summary of the discoveries which are the most important as far as the cellular theory is concerned, and they will easily be able to add their more accurate study of the individual subjects to the connected exposition which I here give them of the whole. Nay, this very

---

* *Cellular Pathology as based upon Physiological and Pathological Histology.* By Rudolf Virchow, Public Professor in Ordinary of Pathological Anatomy, General Pathology and Therapeutics, in the University of Berlin, &c. Translated from the Second Edition of the Original, by Frank Chance, B.A., M.B., Cantab., L.R.C.P., Physician to the Blenheim Free Dispensary and Infirmary. 8vo. London: Churchill. 1860.

exposition may perhaps afford a direct stimulus for such more accurate study; and if it do but this, it will have done enough." (p. xi.)

Again, in the preface to the second edition of the work, the author writes—

"The book will have fulfilled its object, if it assists in the propagation, not of cellular pathology, but, in general only, of independent thought and investigation." (p. xiv.)

In the introduction to the first lecture Professor Virchow throws still more light upon the feelings which governed him in the delivery of the course. He there states that he should "make the attempt to lay before his audience in a more succinct manner the development which he himself, and, as he thought, medical science also, had passed through in the course of the last fifteen years."

The explicit statement of the object, and philosophical estimate of the probable utility, of the work, thus given by the author, are the best indications that can be afforded of its scope and tendency, and it rests for us to sketch the leading characteristics of the theoretical speculations developed in it.

In the application of histology to pathology, "the chief point," writes Professor Virchow, "is to obtain a recognition of the fact, that the cell is really the ultimate morphological element in which there is any manifestation of life, and that we must not transfer the seat of real action to any point beyond the cell." (p. 3.) As a preliminary and essential step towards this end, it is requisite, first of all, that we should have a clear idea of what constitutes a cell. Thus, our author writes:—

"The comparison between animal and vegetable cells, which we certainly cannot avoid making, is in general inadmissible, because in most animal tissues no formed elements are found which can be considered as the full equivalents of vegetable cells in the old signification of the word; and because, in particular, the cellulose membrane of vegetable cells does not correspond to the membrane of animal ones, and between this, as containing nitrogen, and the former, as destitute of it, no typical distinction is presented. On the contrary, in both cases we meet with a body essentially of a nitrogenous nature, and, on the whole, similar in composition. The so-called membrane of the vegetable cell is only met with in a few animal tissues, as, for example, in cartilage; the ordinary membrane of the animal cell corresponds, as I showed as far back as 1857, to the primordial utricle of the vegetable cell. It is only when we adhere to this view of the matter, when we separate from the cell all that has been added to it by an after-development, that we obtain a simple, homogeneous, extremely monotonous structure, recurring with extraordinary constancy in living organisms. But just this very constancy forms the best criterion of our having before us in this structure one of those really elementary bodies, to be built up of which is eminently characteristic of every living thing—without the pre-existence of which no living forms arise, and to which the continuance and the maintenance of life is intimately attached. Only since our idea of a cell has assumed this severe form—and I am somewhat proud of having always, in spite of the reproach of pedantry, firmly adhered to it—only since that time can it be said that a simple form has been obtained which we can everywhere again expect to find, and which, though different in size and external shape, is yet always identical in its essential constituents." (p. 7.)

In such a simple cell dissimilar elements can be distinguished, to wit, (1) a *nucleus*, and, in addition to the nucleus, (2), certain *contents*.

Of the *nucleus* it may be said generally, that "as long as the life of the cell has not been brought to a close, as long as cells contain elements still endowed with vital power, the nucleus maintains a very nearly constant form." Within the nucleus a so-called *nucleolus* is very constantly distinguished in completely developed cells. This structure, however, would not seem to be essential. It has as yet entirely escaped detection in many younger forms, and it would seem "to mark a higher degree of development in the cell."

It is highly probable, Professor Virchow states—

"That the nucleus plays an extremely important part within the cell—a part, I will here at once remark, less connected with the function and specific office of the cell than with its maintenance and multiplication as a living part. The specific (in a narrower sense animal) function, is most distinctly manifested in muscles, nerves, and gland cells; the peculiar actions of which—contraction, sensation, and secretion—appear to be connected in no direct manner with the nuclei; but that, whilst fulfilling all its functions, the element remains an element, that it is not annihilated nor destroyed by its continual activity—this seems essentially to depend upon the action of the nucleus. All those cellular formations which lose their nucleus have a more transitory existence; they perish, they disappear, they die away or break up. A human blood-corpuscle, for example, is a cell without a nucleus; it possesses an external membrane and red contents; but herewith the tale of its constituents, so far as we can make them out, is told; and whatever has been recounted concerning a nucleus in blood-cells, has had its foundation in delusive appearances, which certainly very easily can be, and frequently are, occasioned by the production of little irregularities upon the surface." (p. 10.)

Upon the varying *contents* of the cell, apart from the nucleus, the special peculiarities which individual cells exhibit, under particular circumstances, are in general dependent. "It is not the constituents which we have hitherto considered (membrane and nucleus), but the contents (or else the masses of matter deposited without the cell, *intercellular*) which give rise to the functional (physiological) differences of tissues."

"For us," writes our author, "it is essential to know that in the most various tissues these constituents, which in some measure represent the cell in its abstract form, the nucleus and membrane, occur with great constancy, and that by their combination a simple element is obtained, which, throughout the whole series of living vegetable and animal forms, however different they may be externally, however much their internal composition may be subjected to change, presents us with a structure of quite a peculiar conformation, as a definite basis for all the phenomena of life." (p. 13.)

According to Professor Virchow, this is "the only possible starting-point for all biological doctrines;" and he further argues that, "if a definite correspondence in elementary form pervades the whole series of all living beings, and if in this series something else which might be placed in the stead of the cell be in vain sought for, then must every more highly-developed organism, whether vegetable or animal, necessarily, above all, be regarded as a progressive total, made up of larger or smaller numbers of similar or dissimilar cells." As other corollaries, it is asserted that, "*Every animal presents itself as a sum of vital unities*, every one of which manifests all the characteristics of life," and that "a so-called individual always represents a kind of social arrangements of parts, an arrangement of a social kind, in which a number of individual existences are mutually dependent, but in such a way that every element has its own special action, and, even though it derives its stimulus to activity from other parts, yet alone effects the actual performance of its duties." (p. 14.)

In accordance with this view Professor Virchow has considered it well to "portion out the body into *cell-territories*." In the animal body a peculiarity exists which is rarely found in vegetables, that is to say, the development, in large masses, of so-called *intercellular substance*. Now pathological observation has led our author to the conclusion that the action of cells upon the intermediate substance is definite, and bounded within certain limits—given districts of intercellular matter being ruled over and dependent upon given cells. "But," he continues—

"— there are simple tissues which are composed entirely of cells, cell lying close to cell. In these there can be no difficulty with regard to the boundaries of the individual cells; yet it is necessary that I should call your attention to the fact that, in this case, too, every individual cell may run its own peculiar course, may undergo its own peculiar changes, without the fate of the cell lying next it being necessarily linked with its own. In other tissues, on the contrary, in which we find intermediate substance, every cell, in addition to its own contents, has the superintendence of a certain quantity of matter external to it, and this shares in its changes, nay, is frequently affected even earlier than the interior of the cell, which is rendered more secure by its situation than the external intercellular matter. Finally, there is a third series of tissues, in which the elements are more intimately connected with one another. A stellate cell, for example, may anastomose with a similar one, and in this way a reticular arrangement may be produced similar to that which we see in capillary vessels and other analogous structures. In this case it might be supposed that the whole series was ruled by something which lay who knows how far off; but upon more accurate investigation, it turns out that even in this chainwork of cells a certain independence of the individual members prevails, and that this independence evinces itself by single cells undergoing, in consequence of certain external or internal influences, certain changes confined to their own limits, and not necessarily participated in by the cells immediately adjoining.

"That which I have now laid before you will be sufficient to show you in what way I consider it necessary to trace pathological facts to their origin in known histological elements; why, for example, I am not satisfied with talking about an action of the vessels, or an action of the nerves, but why I consider it necessary to bestow attention upon the great number of minute parts which really constitute the chief mass of the substance of the body, as well as upon the vessels and nerves. It is not enough that, as has for a long time been the case, the muscles should be singled out as being the only active elements; within the great remainder, which is generally regarded as an *inert mass*, there is in addition an enormous number of active parts to be met with.

"Amid the development which medicine has undergone up to the present time, we find the dispute between the humoral and solidistic schools of olden times still maintained. The humoral schools have generally had the greatest success, because they have offered the most convenient explanation, and, in fact, the most plausible interpretation of morbid processes. We may say that nearly all successful practical, and noted hospital physicians have had more or less humoro-pathological tendencies; aye, and these have become so popular, that it is extremely difficult for any physician to free himself from them. The solido-pathological views have been rather the hobby of speculative inquirers, and have had their origin not so much in the immediate requirements of pathology, as in physiological and philosophical, and even in religious speculations. They have been forced to do violence to facts, both in anatomy and physiology, and have therefore never become very widely diffused. According to my notions the basis of both doctrines is an incomplete one; I do not say a false one, because it is really only false in its exclusiveness; it must be reduced within certain limits, and we must remember that, besides vessels and blood, besides nerves and nervous centres, other things exist which are not a mere theatre (Substrat) for the action of the nerves and blood, upon which these play their pranks." (pp. 15, 17.)

After illustrating more fully the essential correspondence which exists between pathological and physiological cells, whether large or small, Professor Virchow enters upon the consideration of physiological tissues. And first he dwells briefly upon the development of cells. Setting aside as no longer tenable the doctrines that living elements, or cells, are "produced from parts previously destitute of shape, such as formative fluids, or matters (*plastic matter, blastema, cytoblastema*)," he asserts that, "even in pathology, we can now go so far as to establish, as a general principle, *that no development of any kind begins* de novo, *and consequently as to reject the theory of equivocal* (spontaneous) *generation just as much in the history of the development of individual parts as we do in that of entire organisms.* . . . . When a cell arises, there a cell must have previously existed (*omnis cellula e cellula*), just as an animal can spring only from an animal, a plant only from a plant." (p. 27.) Hence he arrives at "an eternal law of *continuous development*," whether as applied to entire organisms, vegetable or animal, or the essential constituents of the same.

Now, the normal tissues are susceptible of a very simple classification, dependent upon marked characteristics. There are tissues consisting solely of cells, cell lying close to cell—cellular tissue in the modern sense of the word. To this class belong the epithelial tissues. There are other tissues in which one cell is separated from another by an intermediate substance—the connective tissues. Finally, there is a third class of tissues in which the cells have attained higher and specific forms of development. To this class belong "the nervous and muscular systems, the vessels and the blood." These different classes of tissues having been submitted to a general examination, the question arises how the pathological tissues in their turn comport themselves. To this Professor Virchow replies, that "every pathological structure has a physiological prototype, and that no form of morbid growth arises which cannot in its elements be traced back to some modes which had previously maintained an independent existence in the economy." (p. 60.)

In the development of this proposition it is shown that pathological structures may be classified in a manner similar to that which had already been attempted with physiological tissues. Further, as a consequence of this simple view of the matter, the doctrine of *heterology* of morbid products necessarily falls to the ground. "There is no other kind of heterology of morbid structures than the abnormal manner in which they arise, and this abnormity consists either in the production of a structure at a point where it has no business, or at a time when it ought not to be produced, or to an extent which is at variance with the typical formation of the body." (p. 63.) Again, we are taught, as arising out of this view of the subject, that "no attention is therefore paid, in considering the question of the heterologous or homologous nature of a new formation, to the composition of the structure as such, but only to the relations which subsist between it and the soil from which it springs. Heterology, in this sense, designates the difference of development in the new, as contrasted with the old tissue; or, as we are wont to say, a *degeneration,* a deviation from, typical conformation." (p. 67.) It is manifest that, on this view, the doctrine which declares every pathological new formation to be innocuous which exhibits a reproduction of pre-existing and familiar tissues entirely falls to the ground.

The further development of Professor Virchow's opinions, ranging over the greater part of the pathological field, we shall not attempt to follow, notwithstanding that in so doing we leave not only the greater bulk of his work unnoticed, but also those portions of it which will prove most probably of the greatest interest and moment to the practical pathologist and physician. The nature of his argument renders a profusion of histological and microscopical details necessary for its right comprehension. These cannot well be brought within the compass of a review, or be abstracted without risk to the integrity of the opinions they are designed to support. We have already attempted to set forth the main features of his theoretical speculations, adhering as closely as practicable to the language of the author. We have also briefly indicated the important bearing of these speculations upon one or two pathological doctrines in vogue. To these examples we may fittingly add one or two others taken almost at random from the work, and well calculated, we think, to whet the appetites of our readers for the book itself.

In discussing the influence of the vessels upon nutrition, Professor Virchow writes :—

'If we cut off or diminish the supply of nutritive matter, we must of course prevent the part from absorbing more than its wont, but, *vice versâ,* we cannot, by offering it a larger quantity of nutritive material, straightway compel it to take up more than it did; these are two entirely independent cases. However apt one may be to conclude (and however much I may be disposed to allow, that at the first glance there is something very plausible in such a conclusion) that, from the favourable effect which the cutting off of the supply of blood has in putting a stop to a process which arose from an increase of it, the process depending upon this increased supply, yet I am of opinion that the practical fact cannot be interpreted in this way. It is not so much an increase of quantity, either in the blood as a whole, or in that portion of it contained in an individual part, which is required in order that a like increase should forthwith take place in the nutrition of that part, or of the whole body, as that, in my opinion, particular conditions should obtain in the tissues (irritation) altering the nature of their attraction for the constituents of the blood, or that particular matters should be present in the blood (specific substances), upon which definite parts of the tissues are able to exercise a particular attraction." (pp. 125-6.)

And again he writes, after referring to the specific action of the great secreting organs:—

"Now I demand for cellular pathology nothing more than this view, which must be admitted to be true in the case of the large secreting organs, be extended also to the smaller organs and smaller elements; and that, for example, an epidermis-cell, a lens-fibre, or a cartilage-cell be, to a certain extent, admitted to possess the power of deriving from the vessels nearest to them (not always indeed directly, but often by transmission from a distance), in accordance with their several special requirements, certain quantities of material; and again that, after they have taken this material up, they be held to be capable of subjecting it to further changes within themselves, and this in such a manner that they either derive therefrom new matter for their own development; or that the substances accumulate in their interior, without their reaping any immediate benefit from it; or finally that, after this imbibition of material, even decay may arise in their structure and their dissolution ensue. At all events, it seems necessary to me that great prominence should be assigned to this *specific action of the elements of tissues*, in opposition to the specific action of the vessels, and that in studying local processes we should principally devote ourselves to the investigation of processes of this nature." (p. 129.)

Further we read—

"The greater number of the humoro-pathological doctrines are based upon the supposition, that certain changes which have taken place in the blood are more or less persistent; and just in the very instance where these doctrines have practically exercised the greatest influence, in the theory, namely, of chronic dyscrasiæ, it is usually conceived that the change is continuous, and that by inheritance peculiar alterations in the blood may be transmitted from generation to generation, and be perpetuated.

"This is, I think, the fundamental mistake of the humoralists, the real hinge upon which their errors turn. Not that I doubt at all that a change in the composition of the blood may pertinaciously continue, or that it may propagate itself from generation to generation; but I do not believe that it can be propagated *in the blood itself* and there persist, and that the blood is the real seat of the dyscrasia.

"My cellulo-pathological views differ from the humoro-pathological ones essentially in this, that I do not regard the blood as a permanent tissue, in itself independent, regenerating and propagating itself out of itself, but as in a state of constant dependence upon other parts. We need only apply the same conclusions which are universally admitted to be true as regards the dependence of the blood upon the absorption of new nutritive matters from the stomach, to the tissues of the body themselves also. When the drunkard's dyscrasia is spoken of, nobody of course imagines that every one who has once been drunk labours under a permanent alcoholic dyscrasia; but the common opinion is that, when continually fresh quantities of alcohol are ingested, continually fresh changes also declare themselves in the blood, so that its altered state must continue as long as the supply of fresh noxious matters takes place, or as, in consequence of a previous supply, individual organs remain in a diseased condition. If no more alcohol be ingested, if the organs which had been injured by the previous indulgence in it be restored to their normal condition, there is no doubt but that the dyscrasia will therewith terminate. This example, applied to the history of all the remaining dyscrasiæ, elucidates in a very simple manner the proposition, that *every dyscrasia is dependent upon a permanent supply of noxious ingredients from certain sources.* As a continual ingestion of injurious articles of food is capable of producing a permanently faulty composition of the blood, in like manner persistent disease in a definite organ is able to furnish the blood with a continual supply of morbid materials.

"The essential point, therefore, is to search for the *local origins* of the different dyscrasiæ, to discover the definite tissues or organs from which this derangement in the constitution of the blood proceeds." (pp. 130-1.)

With these illustrations we close this most valuable work. As the author himself states, it contains "no arbitrary settlement of questions, nothing systematical or dogmatical;" hence no juster criticism can be passed upon it, none which redounds more to the honour of its distinguished author, than that the work is in an eminent degree calculated to prove (as he hoped it would prove) "an active ferment to the labourers in the so very various fields of medical science and practice."

---

